# Dielectric Characterization and Separation Optimization of Infiltrating Ductal Adenocarcinoma via Insulator-Dielectrophoresis

**DOI:** 10.3390/mi11040340

**Published:** 2020-03-25

**Authors:** Ezekiel O. Adekanmbi, Anthony T. Giduthuri, Soumya K. Srivastava

**Affiliations:** Department of Chemical and Materials Engineering, University of Idaho, Moscow, ID 83844-1021, USA; adek5632@vandals.uidaho.edu (E.O.A.); gidu3424@vandals.uidaho.edu (A.T.G.)

**Keywords:** dielectrophoresis, electrophysiological properties, crossover frequency, wake or recirculation formation, dielectric spectra

## Abstract

The dielectrophoretic separation of infiltrating ductal adenocarcinoma cells (ADCs) from isolated peripheral blood mononuclear cells (PBMCs) in a ~1.4 mm long Y-shaped microfluidic channel with semi-circular insulating constrictions is numerically investigated. In this work, ADCs (breast cancer cells) and PBMCs’ electrophysiological properties were iteratively extracted through the fitting of a single-shell model with the frequency-conductivity data obtained from AC microwell experiments. In the numerical computation, the gradient of the electric field required to generate the necessary dielectrophoretic force within the constriction zone was provided through the application of electric potential across the whole fluidic channel. By adjusting the difference in potentials between the global inlet and outlet of the fluidic device, the minimum (effective) potential difference with the optimum particle transmission probability for ADCs was found. The radius of the semi-circular constrictions at which the effective potential difference was swept to obtain the optimum constriction size was also obtained. Independent particle discretization analysis was also conducted to underscore the accuracy of the numerical solution. The numerical results, which were obtained by the integration of fluid flow, electric current, and particle tracing module in COMSOL v5.3, reveal that PBMCs can be maximally separated from ADCs using a DC power source of 50 V. The article also discusses recirculation or wake formation behavior at high DC voltages (>100 V) even when sorting of cells are achieved. This result is the first step towards the production of a supplementary or confirmatory test device to detect early breast cancer non-invasively.

## 1. Introduction

Noncommunicable diseases (NCDs) kill more than 36 million people annually representing 63% of global deaths [1]. Breast cancer, a subset of NCDs, accounts for over 500,000 of these deaths [2] with an incidence of about 1.1 million new cases being reported per year [3]. In the United States, as of March 2017, more than 3.1 million women with a history of breast cancer has been reported [4]. About 85% of these breast cancers occur in women who have no family history of breast cancer [4] and one in eight women develop breast cancer in her lifetime. As of now, the main cause of breast cancer cannot be pinned down exactly, but scientists have hypothesized where breast cancer originates. Our bodies consist of many cells, which can be replaced as they age. Old cells tend to copy their DNA before splitting into new ones. However, the copying process could cause mutation which may result in cellular abnormalities called tumors. When tumor cells grow and invade neighboring tissues, they are termed cancerous [5,6]. Breast cancer that starts in the cells of the glands are termed adenocarcinoma (ADCs), which can be invasive ductal (indicates that the cancer cells present in the milk ducts (Ductal Carcinoma in-situ)) and can begin to infiltrate and replace the normal surrounding tissues of the duct walls (accounts for 80% of breast cancer), also known as invasive lobular. According to the National Cancer Institute, around 90% of breast cancers are adenocarcinomas. If untreated, breast cancers can grow bigger, taking over more surrounding breast tissue. When breast cancer cells break away from the original cancer, they can enter the blood or lymph vessels. Traveling through these vessels, cancer cells may settle in other areas of the breast or in the lymph nodes of the breast tissue, forming new tumors. This is called metastasis. These adenocarcinomas are the most difficult tumor to accurately identify the primary site [7].

Diagnosis of adenocarcinoma is achieved by examining features such as tubular myelin, intranuclear surface apoprotein tubular inclusions, Langerhans cells associated with neoplastic cells, cytoplasmic hyaline globules, glycogen, lipid droplets, and cytoplasmic crystals. The diagnostic is painful since a tissue biopsy is utilized to extract the different types of cells mentioned above making it a cumbersome, time consuming, and costly process since they are ultrastructural features that are needed to be observed through electron microscopes. Another technique to diagnose adenocarcinoma is through the application of immunohistochemistry that has also been explored using estrogen and progesterone receptor proteins, thyroid transcription factor-I and surfactant apoproteins [7]. However, specificity and sensitivity are the main issues associated with this method. An alternative technique including a less invasive route is desirable to address some of the drawbacks of the current diagnostic tools used such that it is rapid, easy-to-use, economical, and sensitive. The combination of peripheral blood mononuclear cells (PBMCs) and microfluidics makes an excellent alternative that is explored through this article.

In this article, we explore the use of PBMCs to detect these cancerous cells since they are known to circulate in the peripheral blood of patients, especially when breast cancer is spread beyond the ducts into other parts of the breast tissues or other organs through blood [8]. PBMCs are typically isolated from whole blood using density gradient centrifugation commonly in Ficoll-Pacque PLUS and the Histopaque 1077 media [9]. To discriminate and subsequently separate ADCs from PBMCs, increased interests have been rooted in exploring the utilization of the cell physical properties in lieu of other methods including antibody-conjugation, which is time consuming and can impact ADCs’ properties and viability [10]. Leveraging physical characteristics in the form of size-based filtration [11,12,13], density-gradient separation [14,15,16], and inertial-hydrodynamic discrimination [17,18,19] has been explored in the past until Shim et al. reported that size and density distributions of ADCs tend to overlap with those of PBMCs, leading to occasional inefficiency in the separation of ADCs based on size and density. An alternative technique is to utilize the electrophysiological property differences between PBMCs and ADCs in a microfluidic device, termed as dielectrophoresis (DEP).

Dielectrophoresis (DEP), a microfluidic and an electrokinetic technique that could be utilized to detect ADCs from a heterogenous population of PBMCs, utilizes electric signatures like cell capacitance and conductance in a non-uniform electric gradient, and seems to be a novel alternative [20,21,22,23,24,25]. DEP is a promising technique that is utilized to characterize and manipulate different types of cells like red blood cells, bacteria, virus, yeast, and proteins. It also is capable of detecting subtle changes on the cells based on their state, i.e., alive and dead, healthy and infected. In this article we choose to characterize PBMCs and ADCs utilizing DEP as a detection tool via quantifying the unhealthy or diseased cells, i.e., cancer cells because it employs no moving parts, it is non-destructive for the bioparticles, and utilizes low electric current on a micro-chip without the need of antibody tagging or fluorescent labels making it a portable system. Reduced response time and higher throughput and accuracy makes DEP a promising technique for cancer cell detection.

When a bioparticle is subjected to a non-uniform electric field, the dielectrophoretic forces and the dipole-dipole forces between the particles (dipole moments) are generated based on the differences between the electrical properties (capacitance and conductance) of cells and the surrounding fluid [23]. Traditionally, DEP based cancer cell separations employ metallic electrodes to capture infected cells, by creating non-uniform electric fields using AC voltage [16,25]. AC DEP device offers a major disadvantage in the form of decreased metal electrode functionality due to fouling when biological samples are manipulated [24,26]. Also, these devices often have high cost associated with the fabrication containing metal parts [24,27]. To address the challenges posed by AC DEP device, we chose to explore DC electric current (insulator-based DEP (iDEP)) as an alternative to electrode-based DEP. iDEP employs insulating objects or structures created by microfabrication embedded in the channel to generate spatial non-uniformities in the field [28,29]. With the electrodes placed in the inlet and outlets, here electroosmotic forces can be utilized for inducing flows, eliminating the need for the pumps for continuous operation [30]. The aim of this work, therefore, is to determine experimentally if there are differences in the electrophysiological properties of normal PBMCs and ADCs and to use these differences, if they exist, to numerically attempt their detection (using DC signals) on a microchip—an important step towards the development of a supplementary diagnostic device for ADCs.

**Figure 1:**
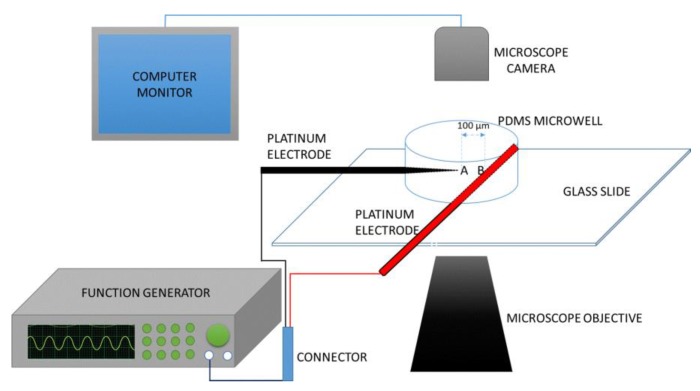
The experimental set-up for the measurement of dielectrophoresis (DEP) crossover frequency using a novel microwell platform to obtain electrophysiological properties, i.e., conductivity and permittivity of peripheral blood mononuclear cell (PBMCs) and adenocarcinoma cells (ADCs) that will aid in designing an early detection platform for breast cancer.

In this article, we develop an in silico based COMSOL Multiphysics model for continuously detecting ADCs from a heterogenous population of PBMCs in a microchannel with modified geometry. To create non-uniformity in the electric field, an array of semi-circular insulating obstacles are embedded in the microchannel. First, a PDMS-based microwell was constructed to house a horizontally arranged 100 µm apart platinum electrode (Figure 1) to obtain the characteristic membrane properties of both ADCs and PBMCs. The properties, validated against the available data in the literature, are then utilized to in conjunction with Finite Element Method (FEM) to model and simulate the trajectory of both cells (ADCs and PBMCs) in a semicircular-insulator-based 2D microfluidic channel. While the characterization of the ADCs gives the innate electrical signatures that is characteristic of moderately differentiated infiltrating ductal adenocarcinoma, the utilization of FEM sets a workable model that could serve as a platform for fabricating a novel diagnostic device for ADCs.

## 2. Theory of Dielectrophoresis

Crossover frequency measurement is a novel dielectrophoretic-based method of characterizing the dielectric properties of many biological particles. By crossover frequency, we mean the frequency at which cells suspended in a microwell in an osmotic concentration medium, change their direction of motion towards or away from the high field region in an electric-field-gradient-based system. When a bioparticle (i.e., cell) is placed between the two electrodes as shown in Figure 1, the cell can either move to A or B depending on its polarizability relative to the medium in which it is suspended. Cells move to A (pDEP) if they are more polarizable than the medium and to B (nDEP) if the reverse occurs. At varying conductivity of the suspending medium, various crossover frequency data (*f*_xo_) can be generated. In this current work, these data are generated, plotted and fitted with a model (Equation (1)) using least square regression and the confidence of the fit was found through the coefficient of regression analysis. A voltage drop at the electrode boundary is considered to be significant at frequencies below 15 kHz [30]. Also, because the reservoirs of microdevice are typically considered as an enormous source of ions compared with the microchannels themselves, any voltage drop can be neglected between the electrode and the inlet or outlet to the microchannel. In this study, the operating conditions for the crossover frequency quantification are maintained above the reported threshold and thus can neglect the voltage drop at the boundary of the electrodes that are placed in the inlet and outlet reservoirs. According to Pethig [31] or a biological cell whose interfacial polarization between the plasma membrane and the cytoplasm results in a dispersion frequency far below 1 MHz for the cell effective dielectric permittivity and conductivity, the first crossover frequency, *f*_xo1_, of the cell membrane is given by:

(1) $$f_{\mathrm{xo}1}=f_{\mathrm{xo}1}(C_{mem},G_{mem},\sigma_{m})\vdots \;f_{\mathrm{xo}1}=\frac{1}{\surd 2}\frac{\sigma_{m}}{\pi RC_{mem}}\sqrt{1-\frac{RG_{mem}}{2\sigma_{m}}-2{\left(\frac{RG_{mem}}{2\sigma_{m}}\right)}^{2}}$$

(2) $$\forall C_{mem}=\varepsilon_{mem}/d$$

(3) $$G_{mem}=\sigma_{mem}/d$$

In terms of total particles and medium properties, $f_{\mathrm{xo}1}$ can also be represented as

(4) $$f_{\mathrm{xo}1}=\frac{1}{2\pi }{\left\{\frac{\left(\sigma_{m}-\sigma_{p}\right)\left(\sigma_{p}+2\sigma_{m}\right)}{\left(\varepsilon_{p}-\varepsilon_{m}\right)\left(\varepsilon_{p}+\varepsilon_{m}\right)}\right\}}^{1/2}$$

where, $C_{mem}$ is the specific membrane capacitance, $G_{mem}$ the membrane conductance, $\sigma_{m}$ the conductivity of the suspending medium, $\varepsilon_{mem}$ the permittivity of the membrane, $\sigma_{mem}$ the conductivity of the membrane, *d* is the characteristic dimension of the cell membrane and R, the radius of the particle. In an iDEP system, the DEP force, ${\vec{F}}_{\mathrm{DEP}}$, acting on the particles due to the field gradient is a function of the particle and medium characteristics and is given as:

(5) $${\vec{F}}_{\mathrm{DEP}}=2\pi \varepsilon_{m}r^{3}\left(\frac{\sigma_{p}-\sigma_{m}}{\sigma_{p}+2\sigma_{m}}\right)\nabla {\left|{\vec{E}}_{DC}\right|}^{2}$$

where the quantity $\left(\frac{\sigma_{p}-\sigma_{m}}{\sigma_{p}+2\sigma_{m}}\right)$ is the Clausius-Mossotti factor (CM), which is the parameter that determines whether ${\vec{F}}_{\mathrm{DEP}}$ will be positive (as in pDEP) or negative (as in nDEP) and $\nabla {\left|{\vec{E}}_{DC}\right|}^{2}$ is the field distribution parameter that enhances particle polarization and dielectrophoretic separation effect.

This force is usually balanced with the viscous drag within the fluid system. Prior to the utilization of DEP-viscous force balance for particle separation, electrokinetic forces (electroosmotic and electrophoretic forces) would have been utilized to pump the particles to the separation region through the electroosmotic channel wall condition and the electrostatic interaction of the electric field with the particles.

Electroosmotic flow is generated due to the action of the electric field on charged interior surfaces having electrical double-layer (EDL). For the microscale flow, surface charge generated at the solid wall-ionic liquid interface is a significant interfacial property to affect the flow. This is because the surface charge at the solid–liquid interface can redistribute the charged ions in the ionic liquid and forms the electrical double layer (EDL) with local net charge density. However, because of the characteristic length of the EDL known as Debye length is small and has the typical values from several nanometers to one micrometer (significantly small compared to the dimensions of the channel), thus the effect of EDL on the microscale flow is usually neglectable and it can only produce obvious effect on the nanoscale fluid flow. When an external electric field is applied on the ionic liquid with EDL within a microchannel, the liquid will be driven by the electric field and form the electroosmotic flow (EOF), which is a typical fluidic transport phenomenon over the microscale [32,33]. The nature and magnitude of the charge in EDL is characterized by the Zeta potential [34]. Electroosmotic mobility of fluid is a function of the Zeta potential of the microdevice, i.e., microchannel construction material and is given by [35]:

(6) $$\mu_{\mathrm{EO}}=\frac{-\xi \varepsilon_{m}}{\eta }$$

where, $\mu_{EO}$ is the electroosmotic mobility,$\varepsilon_{m}$ is the permittivity of medium, ξ is the Zeta potential of the material and η is the viscosity of suspending medium (buffer). The electrophoretic mobility unlike the electroosmotic mobility that depends on the material, depends on the Zeta potential of the particle itself and is given by [36]:

(7) $$\mu_{\mathrm{EO}}=\frac{\xi_{p}\varepsilon_{m}}{\eta }$$

where, $\xi_{p}$ is the Zeta potential of the particle. At the separation region, the particle experiences a dielectrophoretic force that is impacted by the particle mobility. The DEP mobility is a function of CM factor and for a spherical particle it is expressed as [37]:

(8) $$\mu_{\mathrm{DEP}}=\frac{\pi d_{p}^{2}\varepsilon_{m}}{12\eta }CM$$

where, *d*_p_ is the particle diameter and η is the medium viscosity.

## 3. Materials and Methods

### 3.1. Microwell Fabrication

Silicone elastomer mixed with its curing agent in 10:1 ratio (Sylgard 184, Dow Corning, Midland, MI, USA) was placed in a desiccator chamber under 0.27-mTorr vacuum in order to remove the bubbles formed during the mixing process. After three successive degassing operations lasting for 15 min, at an interval of 5 min between each run, the clear PDMS was poured into a clean petri dish and cured in the oven at 70 °C for 1 h. This step was followed by dicing the PDMS into 1” × 1” squares. A 3-mm hole was punched into each of the diced PDMS to create a well onto which the cell suspension was pipetted. Scotch tape was used to remove any dirt/dust from the PDMS after which it is was irreversibly sealed to a borosilicate glass slide through plasma oxidation of 50 W RF power for 1 min. High purity platinum wire was connected to the microwell as shown in Figure 1. With the aid of an Olympus IX71 inverted microscope (Olympus, Tokyo, Japan), the distance (100 µm) between the electrode tips was set. Loctite’s self-mix epoxy was used to keep the electrode spacing intact. The epoxy also prevented any leakage of liquid when the microwell was filled with cell suspension. This was evident when anhydrous copper sulfate was dispensed around the filled microwell, in a regulated environment, did not cause any change in color, i.e., from its natural white to blue color.

### 3.2. Cell Pretreatment

The DEP suspending medium (dextrose solution) was prepared and characterized as described in our previous article [38]. The prepared 100 mL suspending medium was divided into five separate beakers. Into each beaker, except the first, calculated volume of phosphate buffer saline (PBS) was added to successively change the conductivity of the DEP suspending medium to obtain the following conductivities (in mS/m): 50, 60, 74, 88 and 97. Female normal peripheral mononuclear cells (PBMCs) and infiltrating ductal adenocarcinoma cells (ADCs) with no identifiable angiolymphatic invasion were obtained from Conversant Bio, Huntsville, AL, USA. Also, the cells obtained did not have any identifiable information about the patient itself except their gender and age (Institutional Review Board - IRB exempt). The cells were prepared for experiment according to the supplier’s instructions. Thereafter, a known number of cells were transferred into each of the five DEP suspending medium solution where they were washed twice and diluted in 1:400 cell: suspending medium ratio before being pipetted into the DEP microwell for experiment.

### 3.3. Measurement of Crossover Frequency

After the assurance that the microwell was leakage-free, the platinum electrodes were connected to the two terminals of an 80 MHz Siglent SDG 2082X Arbitrary Waveform Generator (Siglent Technologies, Solon, OH, USA), which supplied an 8 V peak-to-peak sinusoidal AC signal of shifting frequencies. 5 µL of the PBMCs suspension prepared as discussed in Section 3.2 was transferred into the microwell and allowed to equilibrate. Then, about 4 µL was carefully siphoned from the well so that fewer cells (between 4–7 cells) were present in the field-of-view for the experiment. Having fewer cells does not only reduce the influence of particle-particle interaction, but also enhances clarity in visualizing cells for crossover frequency determination. The waveform generator was then switched on to generate electric field gradient around the electrodes. Movement of cells was monitored and captured with a high-speed camera at 30 fps as a function of the changing field frequency until the crossover frequency was found. There was no movement of cells or flow when the waveform generator was turned off. The experiment was repeated four times (technical replicates) and there were two set of biological replicates obtained for the experiments and the crossover frequency was found in each case. More experiments were run using the other modified medium at varying conductivities thus obtaining crossover frequency spectra. The PBMCs are majorly lymphocytes (>80%) ~10 µm in diameter while ADCs are ~20 µm in diameter. Measurements were made at room temperature conditions, i.e., *T* = 24 ± 1 °C.

## 4. Finite Element Modeling and Simulation

**Figure 2:**
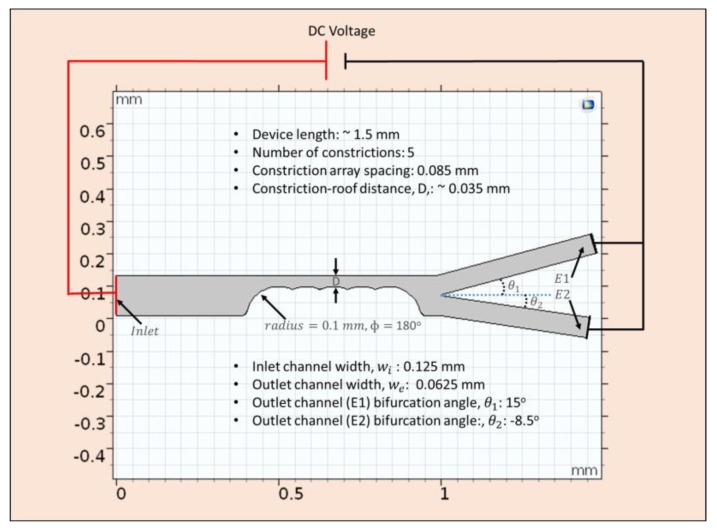
Optimal device design geometry obtained by COMSOL modeling and simulation utilizing the electrophysiological properties of PBMCs and ADCs from the PDMS microwell. Entire microfluidic platform is ~1.5 mm with semi-circular constrictions embedded in the channel. Inlet channel is 125 μm wide and the two outlet channel widths are ~62.5 μm. Pt electrodes in the inlet and outlet ports is connected to a DC power supply to further sort ADCs from healthy PBMCs.

In this section, attention was given to the numerical modelling and simulation performed using the dielectric properties obtained from the crossover frequency measurements in Section 3.3. COMSOL Multiphysics 5.3a (Comsol Inc., Stockholm, Sweden) was used to solve fluid flow, electrostatics, and particle tracing modules in an integrated stationary and time-dependent fashion. The architecture of the separation device (Figure 2) was arrived at after a series of parameter modification that ensured a complete separation of ADCs from its mixture with PBMCs. The design was made in 2D because the width to depth ratio was more than 5:1.

Electric current mode was used to solve, in steady states, the current conservation equation based on Ohms law and electric displacement relations using the electric potential as the dependent variable. This was solved in steady state because the charge relaxation time for the conducting media (water, in this case 3.6 × 10^−6^ s) is less than the external time scale for device operation (10 s). Solving this Ohm’s law with the charge conservation and electric displacement equations gives the electric field, E, which was used to compute the electroosmotic boundary condition used in the creeping flow analysis according to $u_{\mathrm{EO}}=\mu_{\mathrm{EO}}E$, where $u_{\mathrm{EO}}$ is the electroosmotic velocity- the velocity of the bulk of the fluid flowing in the channel due to electric field effects. In the incompressible creeping flow analysis (as is the usual case in microfluidic channel where the Reynolds number is significantly less than unity and viscous force is dominant), the steady state form Stokes equation together with the continuity equation was solved as the synergistic conservation of momentum and mass, which account for the velocity profile within the fluidic channel. The magnitude of this velocity as a function of the position within the channel was then used to solve the drag force (Table 1) acting on the particles flowing within the channel through numerical coupling. The drag force is then counterbalanced by the dielectrophoretic force, Equation (5), at the region where the magnitude of electric field norm was modified as a result of the constrictions placed between the inlet and the outlet channels. Each particle moving through the microdevice was tracked using the particle tracing module solved in time-dependent mode. Tracking the particles enabled the statistics through which the device parameters (like voltage, device dimensions, etc.) that generated the desired separation of the particles were noted. Using a free triangular customized mesh, with different sizes, growth rate, and curvature factor for both constrictions and the remaining regions within the channel, the geometry was discretized and made ready for finite element analysis. Multifrontal massively parallel (MUMPS) solver, which performs Gaussian factorization, was used in the stationary mode to obtain the velocity profile and the electric field norms. MUMPS solved for the velocity profile and the electric field norms these values with a relative tolerance of 0.001 and without any recourse to lumping while computing the fluxes. GMRES (generalized minimal residual solver), a solver that approximates solutions by the vector in a Krylov subspace with minimal residual, was used in the transient domain to track the particles with respect to their position in space and velocity magnitude as a function of time. The final geometry of the device where complete separation occurs was 1.4 mm in length with 5 semi-circular constriction of radius 0.1 mm and inter-structural constriction spacing of 85 µm. The distance *D* (Figure 2) between the constriction end and the upper channel wall was fixed to be 35 µm. This distance forbids two cancer cells to pass through the separation region at any given time. This design was made to prevent any form of shielding that may eventually result in incomplete separation. Table 1 provides a list of parameters, variables, boundary conditions in each type of study utilized in COMSOL, and equations associated with the modeling and simulation. Zeta potential for PDMS was assumed to be −0.1 V and a relative permittivity of 80 was used in the simulation. The flow velocity at the inlet was assumed to be 0.001 m/s.

## 5. Results and Discussion

In this section, we finally discuss and present the important results to prove that breast cancer can be detected early enough using whole blood. This simulation study demonstrating sorting of ADCs from PBMCs will further be validated experimentally (beyond the scope of this article). Our results are categorized into sections demonstrating: (1) experimental evidence of electrophysiological characterization of both healthy PBMCs and breast cancer ADCs, (2) validation of our microwell technique by comparing with studies from literature and (3) modeling and simulation parameters like meshing, stationary analysis, transient analysis.

### 5.1. Electrophysiological Characterization of PBMCs and ADCs Experimentally

**Figure 3:**
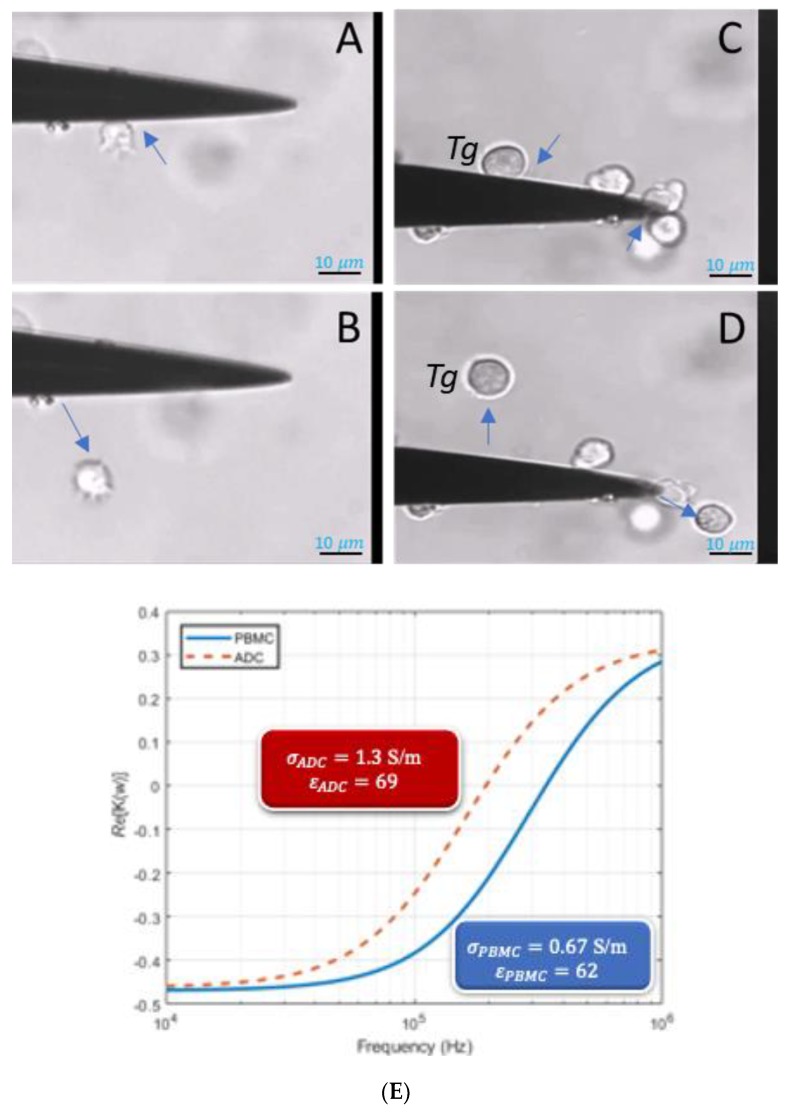
ADC cells experiencing DEP in the microwell at varying AC frequencies- (**A**) shows the ADC cells experiencing positive DEP (pDEP) wherein the cells move towards the high-field region or the triangular electrode in here and (**B**) shows the ADC cells experiencing nDEP behavior wherein the cells move away from the high field region. (**C**,**D**) are the images resulting from manual tracking of the target cell (labeled Tg) as demonstrated in [39]. (**E**) shows a plot of real part of Clausius-Mossotti factor varying with frequency (Hz). Here the cells initially experience nDEP (negative DEP) and as the frequency increases, they switch to pDEP (positive DEP) where the switch is termed as first crossover frequency.

In estimating the properties of both PBMCs and ADCs movement of cells toward or away from high field region was tracked until the crossover frequencies were found in case at changing properties of the suspending medium. Figure 3A,B shows an ADC cell experiencing positive and negative DEP force (pDEP and nDEP) respectively. In Figure 3C,D, we manually tracked the movement of the target cell as previously demonstrated for prostate cancer in Hele-Shaw flow cell by Huang et al. [39]. Figure 3E shows the first crossover frequency behavior when cells experience a switch from nDEP to pDEP. This first crossover frequency, usually in Hz–kHz range is mainly due to the membrane associated proteins, shape, and size of the cell. The first crossover frequency is often enough to study the phenotype of the cells; however, to characterize their genotype, the 2nd crossover frequency value has to be obtained, often in MHz range.

The crossover frequency data for PBMCs and ADCs were fitted using Equation (1). This equation is an ideal model owing to the spherical nature of lymphocytes. Equation (1) is also suitable for ADCs even though they are a little distorted. Kirby and Huang et al. had reported that an isotopically inhomogeneous non-spherical cell can still be analyzed, to a good approximation, with single shell model since the spherical harmonic solutions used in the eigenfunction expansion approximations for DEP force can help define the effective particle properties [39,40]. Since the second crossover frequencies could not be obtained due to the limitation of the measuring equipment, often obtained at high frequency range (>50 MHz), the cytoplasmic properties used in the simulation were as reported by Qiao et al. using impedance measurement [41]. The total (effective) particle conductivity, σ_p, and permittivity was obtained as described by Adekanmbi et al. [3] and Pethig [31] respectively. Table 2 provides the dielectric properties obtained from our experiments that are compared to the other published literature values in case of PBMCs. It should be noted that the conductivity of the infected ADCs rise sharply compared to the healthy PBMCs that may be attributed to the introduction of new membrane permeation pathways, to membrane peroxidation damage, and to changes in membrane fluidity following infection [42].

The data fitted using MATLAB in Figure 3E were analyzed using chi-square test. The expected value of the crossover frequency is determined from the curves in Figure 3E at *Re* (K(w)) = 0 to prove that the fitting is reasonable. The $\chi^{2}$ critical value at 0.05 significance value is obtained to be 11.07. The $\chi^{2}$ test statistic value for ADCs and PBMCs are found to be 1.1804 and 2.413 respectively. Since, the calculated test statistic is less than the critical $\chi^{2}$ value, it signifies a reasonably good fit, i.e., there is no significant difference between the observed (curve) and expected (experimental) values.

However, the authors believe that this is the first time that ADCs were characterized using a novel electrokinetic technique to obtain their dielectric properties i.e., permittivity and conductivity as shown in Table 2.

### 5.2. Parameters Affecting COMSOL Modeling and Simulation to Obtain High Sorting Efficiencies

In this section, we discuss the factors that affect the optimization of the device geometry to achieve high sorting efficiencies that further influence early breast cancer detection obtained through sorting ADCs from healthy PBMCs.

#### 5.2.1. Meshing of the Device Design in COMSOL

**Figure 4:**
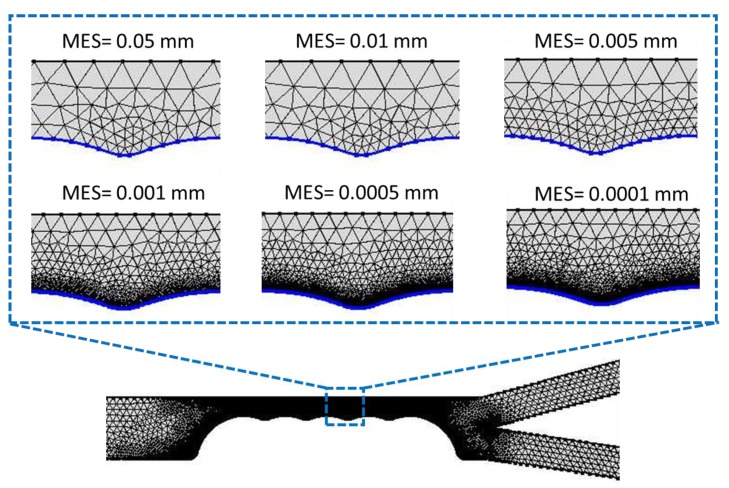
The discretization of the separation region, i.e., along the semicircular constrictions where maximum DEP effect is observed that causes the cells to move into categorized streamlines by adopting variable mesh element size (MES).

Meshing is one of the factors that strongly affect modelling requirements. Choosing the right mesh element types and sizes is highly pivotal to the accuracy of the simulation results in any finite element problem. Under-meshing can result in solutions that are far less than accurate while over meshing can result in large amount of computational time due to the mesh using too many unnecessary elements. To prevent under meshing, we used mesh elements greater than 40,000. Over meshing was, however, checked and prevented by using meshing sequence with local and global attributes. The local mesh density at the constrictions was sufficiently increased by reducing mesh size while the remaining part of the geometry (where dielectrophoretic force would not have significant effects) was meshed at increased mesh size. This meshing sequence, which was based on Lagrange quadratic representation, reduced the total number of mesh element by 45.17% and computation period by 51.06%. Since the dielectrophoretic force, which causes cells to separate based on their movement away or towards the high field region, acts significantly at the channel constriction zone, it was necessary to verify if the maximum element size (MES) at the constriction would affect the transmission probability of ADCs and to what extent would that effect be. As shown in Figure 4, the accuracy of the solution (which is a function of the transmission probability) depends on the choice of mesh size. The mesh characteristics that was found to be optimum at the applied effective potential difference is as given in Table 1.

**Figure 5:**
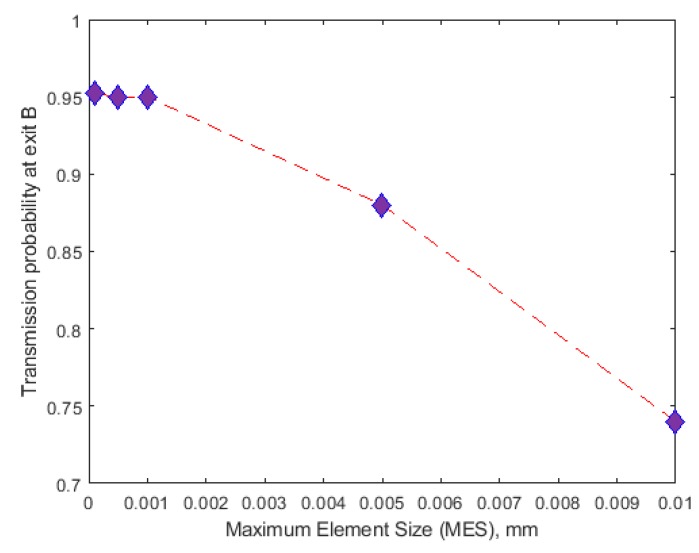
Transmission probability of ADCs as a function of the maximum element size at the separation zone, i.e., around the semicircular constriction region. Since MES at 0.001 mm, 0.0005 mm, and 0.0001 mm are almost similar, the simulation was completed fixing MES at 0.001 mm.

Figure 4 demonstrates the gradation of the discretization regime of the constriction zone as the maximum element size (MES) is progressively reduced. At MES = 0.05 mm the density of the triangular mesh is very low indicating that the inter-nodal distance within the discretized zone is large. This large distance depicts an inefficient solution capacity for the gradient of the electric field, which is evident in the low transmission probability (TP) for the ADCs (Figure 5). Transmission Probability, TP, is referred to as the ratio of the number of particles at a specified exit to the total number of the particles at the inlet. In other words, large mesh size at the constriction zone was not able to correctly solve for the electric field gradient which is necessary for dielectrophoretic influence on the particles. When the mesh size was progressively reduced, the number of elements increased correspondingly. This in turn increased the separation efficiency at the constriction zone, hence the dramatic ramping up of the percentage of ADCs that were sorted from healthy PBMCs. It is important to note that the progressive reduction in MES increases the computation time, i.e., with MES at 0.0005 mm and 0.0001 mm requiring 800% and 960% more time than at 0.001 mm. Since the TP value for MES = 0.001 mm, 0.0005 mm and 0.0001 mm are comparatively similar and >97%, the computation was carried out at 0.001 mm with an error margin of ~<0.00020%. At these values (0.001, 0.0005 and 0.0001 mm), it is safe to conclude that the stationary and transient solutions (within the margin of error) of the coupled physics do not vary with mesh condition as the TP values tend to be approximately constant. MES value beyond 0.0001 mm showed critical error warning sign in COMSOL and was computationally very expensive.

#### 5.2.2. Stationary Field Analysis

The numerical computation comprises of two stationary fields: (a) creeping (fluid) flow and (b) electric current (ec). The electric current was solved in the stationary mode to generate the electric field that not only generated the electro-osmotic effects at the channel wall but also provided the distribution of field strength, E, whose gradient provided the necessary dielectrophoretic force at the constrictions.

**Figure 6:**
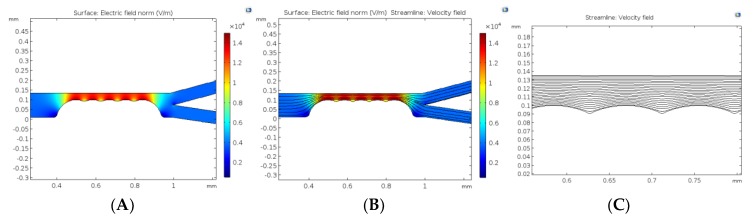

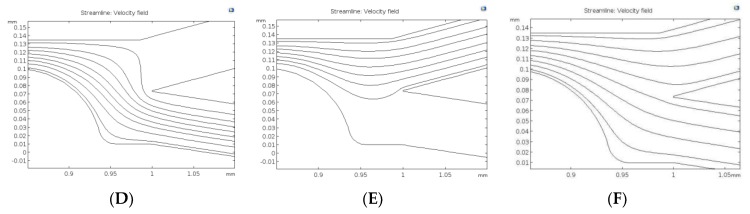
Field and velocity profiles obtained from solving the electrostatics and stokes equations in stationary mode. (**A**) is the electric field norm, (**B**) is the combination of the field norm with velocity streamlines, (**C**) is the zoomed image showing the effect of the constriction zone on velocity streamlines. (**D**–**F**) show streamlines based on changing DC voltage at 10 V, 110 V and 60 V respectively. The constriction diameter and number were fixed to be 100 μm and 5 respectively.

As shown in Figure 6A, when DC potential difference was applied across the channel (from the inlet to outlet) and there was a distribution of the electric field as governed by the Laplace equation. The tips of the constrictions within the channel, generated the highest field strength region that was important for dielectrophoretic separation. In Figure 6, the effect of the field gradient is visually more pronounced when the flow field lines were plotted together with the electric field norm. Glaringly, the gradient of the field which was utilized by the dielectrophoretic force acting at the constrictions interfere with the velocity field. The dielectrophoretic velocity introduced at the constrictions added to the already existing electrophoretic and electroosmotic velocities apart from the increase in velocity that was introduced by the continuity equation owing to the reduction in flow area. Figure 6C shows the ripple effects generated from the surface of the constrictions outwards. The resultant effects of this streamline interference could be seen in Figure 6D–F at varying DC voltage, i.e., 10 V, 110 V, and 60 V respectively. The number of constrictions were fixed at 5 and diameter of the constriction was considered to be 100 μm.

**Figure 7:**
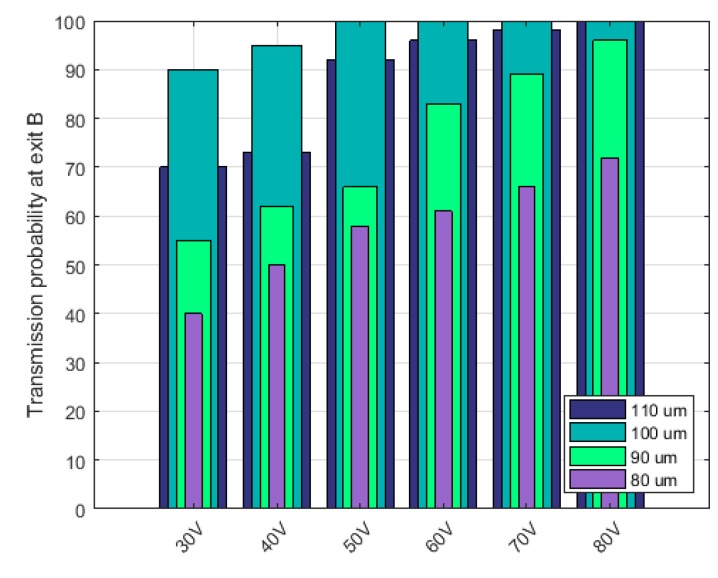
Effects of constriction clearance / size, i.e., diameter and DC voltage on the transmission probability of ADCs. Diameter of the constrictions were varied—80, 90, 100, and 110 μm keeping the number (#) of constrictions fixed, i.e., 5 at a given time. Perfect sorting was observed for constriction diameter of 100 μm at voltages >50 V_DC_.

Effects of applied potentials and constriction radius on transmission probability: It is important to verify the effects of the electric field strength at various applied potentials and constrictions on the separation efficiency of the microdevice platform. As a result, the radius of the constrictions was varied keeping the number (#) of constrictions fixed at 5 at a given time thus resolving the Laplace equation each time using different applied potential without varying the mesh conditions (Figure 7). The number of constrictions was fixed as an optimization test constraint with respect to the length of the device as well as the exploration of the possibility of initiating cellular separation with minimal insulating constrictions as previously demonstrated by Adekanmbi et al. [38].

Transmission probability of the total number of PBMCs (from inlet to outlet) as a function of the constriction gap, i.e., D in Figure 2 and applied voltage using a fixed number of constriction entities, i.e., 5 was calculated and plotted as shown in Figure 7. Transmission probability (TP) is congruent to normalizing the amount of PBMCs recovered by the initial amount of PBMCs in the inlet mixture. This means, a TP value of 1 represents 100% separation of the PBMCs from its mixture with ADCs. The essence of calculating the transmission probability was to verify the selectivity of the device and to track operating parameters that would be optimal for the operability of the device. Figure 7 demonstrates the effect of varying the constriction diameter and the applied voltage on the transmission probability of PBMCs. The TP value for each of the constriction diameter was progressively increased with the changing potential. At a given DC voltage, the recovery of PBMCs was highest when the constriction diameter was 100 μm. More so, from 50 V to 80 V, 100 μm constriction diameter gave a perfect separation of the PBMCs. None of the constriction diameters gave 100% separation except 110 μm at 80 V. These variations in transmission probability could be attributed to the changing electric field strengths as the applied voltage and constriction diameter change. Changing the applied DC potential affected the electrokinetic and dielectrophoretic forces operating within the channel. Electrokinetic contributions within the channel affects the particle velocity and hence the resident time within which the particles are expected to experience strongest DEP force at the constriction zone. At <50 V, the electro-osmotic velocity of the bulk fluid medium was low enough to move the particles slowly to the constriction zone where there is ample residence time for the cells to experience sufficient induced dielectrophoretic force that would cause them to be separated adequately. However, since DEP force depends on square of the field gradient, the low DC potential (<50 V) could not generate the required electric field gradient that is sufficient to induce strong negative dielectrophoretic force necessary for sorting the cells into their respective differential outlets.

Since increasing electric potential could result in increased Joule heating of the microdevice leading to unwholesome modification of the electrokinetic and dielectrophoretic effects, 100 μm constriction diameter was considered to be the ideal dimension for the separation device platform. Furthermore, operating at a lower voltage, i.e., ~60 V_DC_ seems to reduce the risk of Joule heating within the insulator-based dielectrophoretic device.

#### 5.2.3. Transient Analysis

Particle tracking analysis was used to trace the movement of both ADCs and PBMCs along the whole microdevice platform. There is no change observed in the particles’ trajectory when the particle position is placed either at the center or the edge of the inlet, since a uniform particle distribution is selected in COMSOL within the channel. There is no surface interaction with the particles due to the “bounce-off” condition selected in COMSOL. Particles were seen moving through the channel inlet until they were acted upon by the dielectrophoretic force at the constriction which tend to move the particles either towards or away from the constriction surface depending on the properties of the medium, ADCs, PBMCs, and the generated electric field gradient. From the equation of the dielectrophoretic force (Equation (5)), the force experienced by both PBMCs and ADCs at the constriction depends on the square of the electric field gradient as well as the radius of the particle to the third power. The membrane conductivity of both ADCs and PBMCs are both less than that of the medium. Therefore, it is expected that both of them would experience negative DEP.

**Figure 8:**
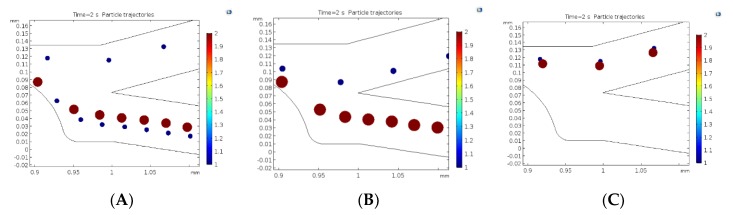
Particle trajectories showing partial (**A**,**C**) and complete separation (**B**) at various voltage conditions. The constriction diameter was fixed at 100 µm along with number of constrictions at 5. (**A**) shows incomplete separation at <50 V_DC_, (**B**) complete separation at 50 V_DC_, (**C**) no separation at >100 V_DC_.

**Figure 9:**
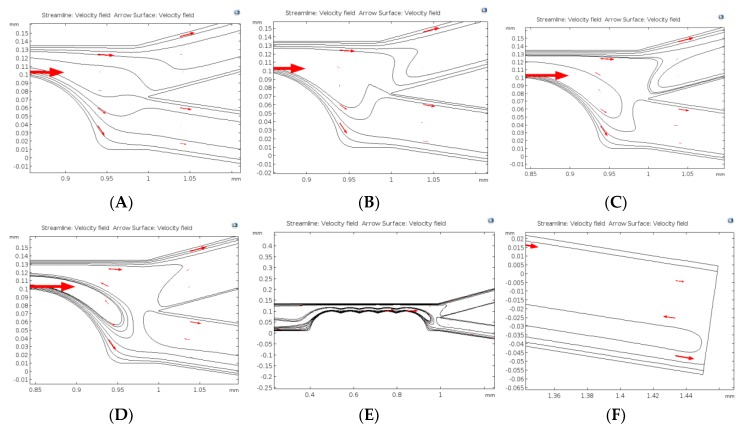
Velocity streamlines at various applied DC voltage conditions at fixed number of constrictions (5) and constriction size (100 µm); (**A**) and (**B**) shows streamlines at 60 V_DC_ and 100 V_DC_ respectively; (**C**) partial recirculation observed at 120 V_DC_; (**D**) increasing recirculation at 200 V_DC_; (**E**) shows the recirculation effects that caused non-compliant behavior of cells to the DEP force; and (**F**) close-up of some cells that tend to reach the exit showing recirculation as well.

Figure 8 demonstrates the scenario where ACDs and PBMCs were partially and completely separated while shifting the applied voltage for a constriction number of 5 and diameter of 100 µm at fixed medium properties. Figure 8A depicts incomplete separation at DC voltage below 50 V. At this applied voltage the strength of the applied electric field was not sufficient enough to push away the PBMCs. At 50 V, the generated field gradient had made the DEP force more negative such that the PBMCs were pushed away enough from the high field region to cause their separation from ADCs (Figure 8B). No separation was observed at higher DC voltages, i.e., >100 V (Figure 8C). However, there was separation at ~100 V but with some interesting modification to the flow streamlines as shown in Figure 9.

In Figure 9A, the operating DC voltage is 60 V while Figure 9B is at 100 V. At 120 V (Figure 9C), some streamlines are being recirculated and this recirculation became more and more pronounced as the applied voltage increased to 200 V (Figure 9D–F). Figure 9D shows the close-up section of the bifurcation zone at 200 V, where in more recirculation of streamlines are being observed. Figure 9E is a close-up representation of the streamline recirculation showing some of the cells that were forced to move in a circular reverse direction to the DEP force. As shown in Figure 9F, some of the cells that were heading towards the exit ports are also forced to move towards the inlet, i.e., recirculated back into the main channel.

This interesting development may be associated with the inter-relation of the high potentials with the electric current within the channel, which, could generate a substantial amount of temperature rise that interferes with the conductivity of the fluid-particle system. Since dielectrophoretic force is a function of conductivity of the fluid-particle system, DEP is hampered as Joule heating becomes more predominant due to the increased temperature. This simulation neglected particle-particle interaction on the basis of experiments since the cell suspension were to be diluted to an extent where in the cells will substantial be far apart that their interaction can be considered inconsequential.

### 5.3. Validation of the DEP Microwell Technique

**Figure 10:**
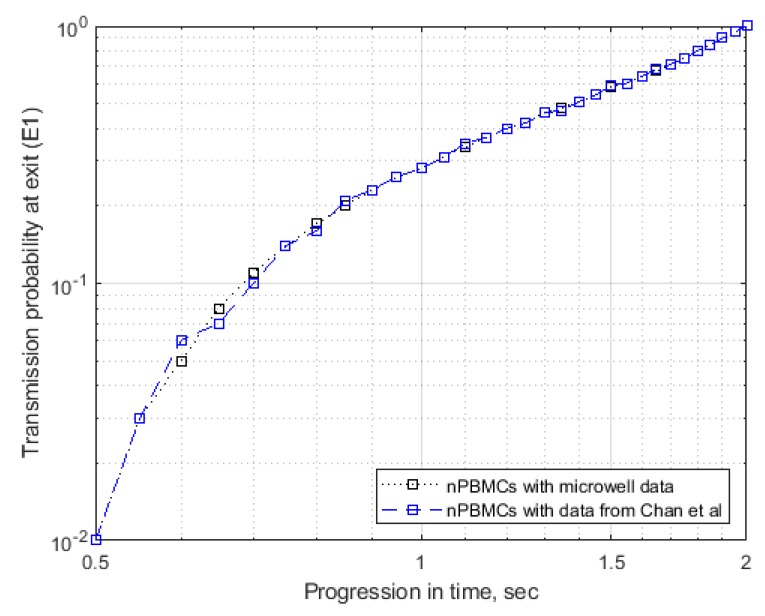
Validation of the electrophysiological properties of healthy PBMCs obtained from the DEP microwell platform using crossover frequency measurement and the literature reported values based on electro-rotation experiments [43]. Both the samples were derived from non-pregnant young women (<50 years of age).

The electrophysiological properties for PBMCs obtained data through our novel microwell platform via DEP crossover frequency measurement were validated by comparing the reported data in literature obtained through DEP electro-rotation measurement as reported by Chan et al. [43]. The samples used in this research and the reported literature values were from non-pregnant young female (<50 years of age) since pregnancy tends to substantially lower the specific membrane conductance of PBMCs [43]. The electrophysiological properties for PBMCs obtained from our experiments and the literature reported values were used to run the simulation independently under the same operating conditions. Figure 10 shows the results of the comparative simulation where a log-log plot of transmission probability (TP) and the progression time were plotted. Statistically, the p-value obtained for this comparison was 0.1 (at 0.01 significance level) implying that we do not have sufficient evidence to reject the null hypothesis of “no significant difference” between the two outcomes.

## 6. Conclusions

Continuous dielectrophoretic separation of infiltrating ductal adenocarcinoma cells (ADCs) from isolated peripheral blood mononuclear cells (PBMCs) using direct current in a semi-circular insulator-based microchannel has been numerically studied. The electrophysiological properties for PBMCs used in simulations were obtained in a novel DEP microwell platform that characterized the behavior of the cells under varying AC frequency by measuring the DEP crossover of the cells. The first and second crossover frequency obtained were curve-fitted using a single shell model to obtain the conductivity and permittivity of the PBMCs. PBMCs vary in size and the sample used in this experiment was majorly small size lymphocytes, i.e., ~10 μm in diameter.

Dielectrophoretic force is a function of the gradient of the electric field-a factor that also depend on the applied voltage as well as the constriction radius. In order to induce sufficient field gradient for the dielectrophoretic separation of ADCs from PBMCs, the applied DC potential and the constriction diameter were dynamically varied until a regime of perfect simulation was obtained at constriction diameter of 100 μm and applied DC potential of ~50 V. The number of constrictions in the channel also affects separation efficiency and 5 semi-circular constrictions lead to optimal sorting of ADCs from PBMCs. This insulator-DEP based method of separating infiltrating ductal adenocarcinoma cells (ADCs) from isolated peripheral blood mononuclear cells (PBMCs) using direct current provided a cheaper, less cumbersome, easier-to-use, and yet efficient approach when compare with previous methods that used deformability, magnetism, or dielectric affinity column. Discretization of domain (meshing) in numerical analysis is an important factor that affect the accuracy of the solution obtained from solving any associated physics within a microchannel. In this paper, effort was made to strike a balance between the computational requirements of the mesh size and the desired transmission probability. Mesh size (local and global) was carefully chosen such that the resultant solution of the simulation did not vary with meshing. To aid our understanding of the choice of applied potential and how it relates with the separation efficiency, we found out that increasing the voltage beyond 100 V DC would lead to no separation, i.e., both PBMCs and ADCs moved into one exit channel.

Another interesting phenomenon was observed at higher voltages (>100 V) along with separation was recirculation behavior of cells. Some of the cells that were moving towards the exit channels were forced to change their direction back to the inlet. Recirculation increased especially between the constriction region and the bifurcation into exit channels with increasing DC potential. This behavior or wake formation is due to increased Joule heating as the temperature rises in the microfluidic platform with increasing DC potential since DEP is a function of the conductivity of the medium.

This DEP spectroscopy technique based on crossover measurement allows characterizing the intracellular differences and physical properties of cells, without any labeling, without affecting cell integrity and viability. Finally, this method confirms a high potential of emerging lab-on-chip (LOC) platforms in the early diagnosis and the treatment of breast cancer especially in young women where mammography is ineffective and/or painful.

## Figures and Tables

**Table 1:** List of parameters, variables, discretization, type of study utilized, and the equations associated that was incorporated into COMSOL package for optimizing the device geometry and sorting of adenocarcinoma (ADCs) from healthy peripheral blood mononuclear cells (PBMCs).

Physics/Parameters	Tag	Dependent Variable	Discretization	Study	Equation
Electric current	ec	*V*	Lagrange Quadratic	Stationary	$\nabla \cdot J=Q_{j\cdot v}$ $J=\sigma E+J_{e}$ E=−∇V Wall boundary- insulated (n·J=0)
Fluid Flow	spf	*u*	P2 + P1	Stationary	$\nabla \cdot \left[-pI+\mu (\nabla u+{\left(\nabla u\right)}^{T}\right]+F=0$ ρ∇·(u)=0 Wall boundary- electroosmosis $u=\mu_{eo}E_{t}$ $\forall \;\mu_{eo}=\frac{\epsilon_{r}\epsilon_{0}}{\mu }\xi ;E_{t}\;=E-\left(E\cdot n\right)n$
Particle tracing	ptf	*q*, *v*	Formulation	Transient	$\frac{d\left(m_{p}v\right)}{dt}=F_{t}$ $F_{D}=\frac{1}{\tau_{p}}m_{p}\left(u-v\right)$ $\tau_{p}=\frac{\rho_{p}d_{p}^{2}}{18\mu }$ $F_{DEP}\;=2\pi r_{p}^{3}\epsilon_{0}real\left(\epsilon_{r}^{*}\right)real\left(K\right)\nabla {\left|E\right|}^{2}$ $K=\frac{\epsilon_{r,p}^{*}-\epsilon_{r}^{*}}{\epsilon_{r,p}^{*}+2\epsilon_{r}^{*}}\forall \epsilon_{r}^{*}=\epsilon_{r}$ in stationary field Wall boundary- particles bounce-off walls
			Newtonian		
			Drag law		
			Stokes		
Meshing	**Calibration**		**Mesh Type**	**Max size**	**Boundary layer transition**
	Fluid dynamics		Free triangular	0.001 mm	Smooth transition to interior mesh
Stationary solver	MUMPS				
Transient Solver	GMRES				

**Table 2:** Dielectric properties, i.e., conductivity and permittivity obtained from our novel electrokinetic technique based on cell response obtained at crossover frequency for ADCs and healthy PBMCs using an osmotic concentration suspending medium maintained at osmotic conductivity and permittivity. Literature reported values has been compared with our novel technique for PBMCs only as a measure of validation [43].

Property	ADCs (Infiltrating Ductal Adenocarcinoma Cells)	PBMCs (Lymphocytes)		Suspending Medium
	Crossover Freq. Technique	Crossover Freq. Technique	Literature Reported [43]	
Conductivity (S/m)	1.3	0.67	0.66	0.055
Permittivity	69	62	59.62	80
